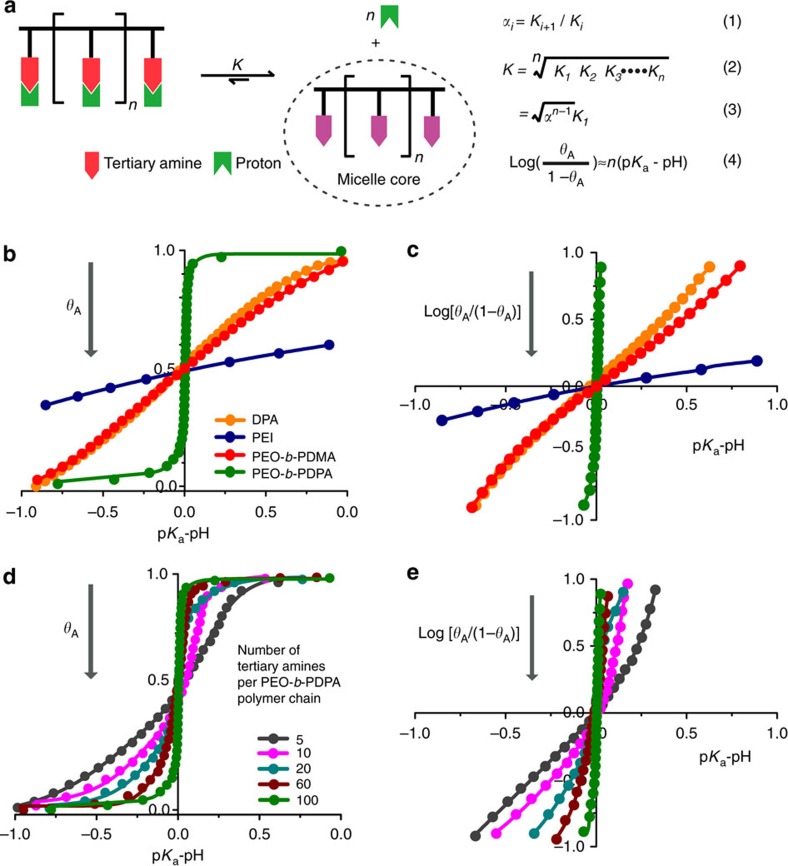# Erratum: Molecular basis of cooperativity in pH-triggered supramolecular self-assembly

**DOI:** 10.1038/ncomms13777

**Published:** 2016-12-16

**Authors:** Yang Li, Tian Zhao, Chensu Wang, Zhiqiang Lin, Gang Huang, Baran D. Sumer, Jinming Gao

Nature Communications 7: Article number: 13214; DOI: 10.1038/ncomms13214 (2016); Published: 10 27 2016; Updated: 12 16 2016

Figure 5 of this article contained errors introduced during the production process. In Fig. 5c, the minimum value of the *x* axis (left hand side) should be −1.0 and not 1.0. Additionally, the maximum value of the *y* axis in Fig. 5d should read 1.0 instead of 0.6. The corrected version of Fig. 5 is shown below as Fig. 1.

## Figures and Tables

**Figure 1:**