# Supplementary material for: Molecular basis of cooperativity in pH-triggered supramolecular self-assembly
Source: Nat Commun. 2016 Oct 27;7:13214. doi: 10.1038/ncomms13214 (PMC5095283; doi:10.1038/ncomms13214)
Supplement: Supplementary Information — Supplementary Figures 1-17, Supplementary Tables 1-2, Supplementary Discussion, Supplementary Methods and Supplementary References. [file ncomms13214-s1.pdf]

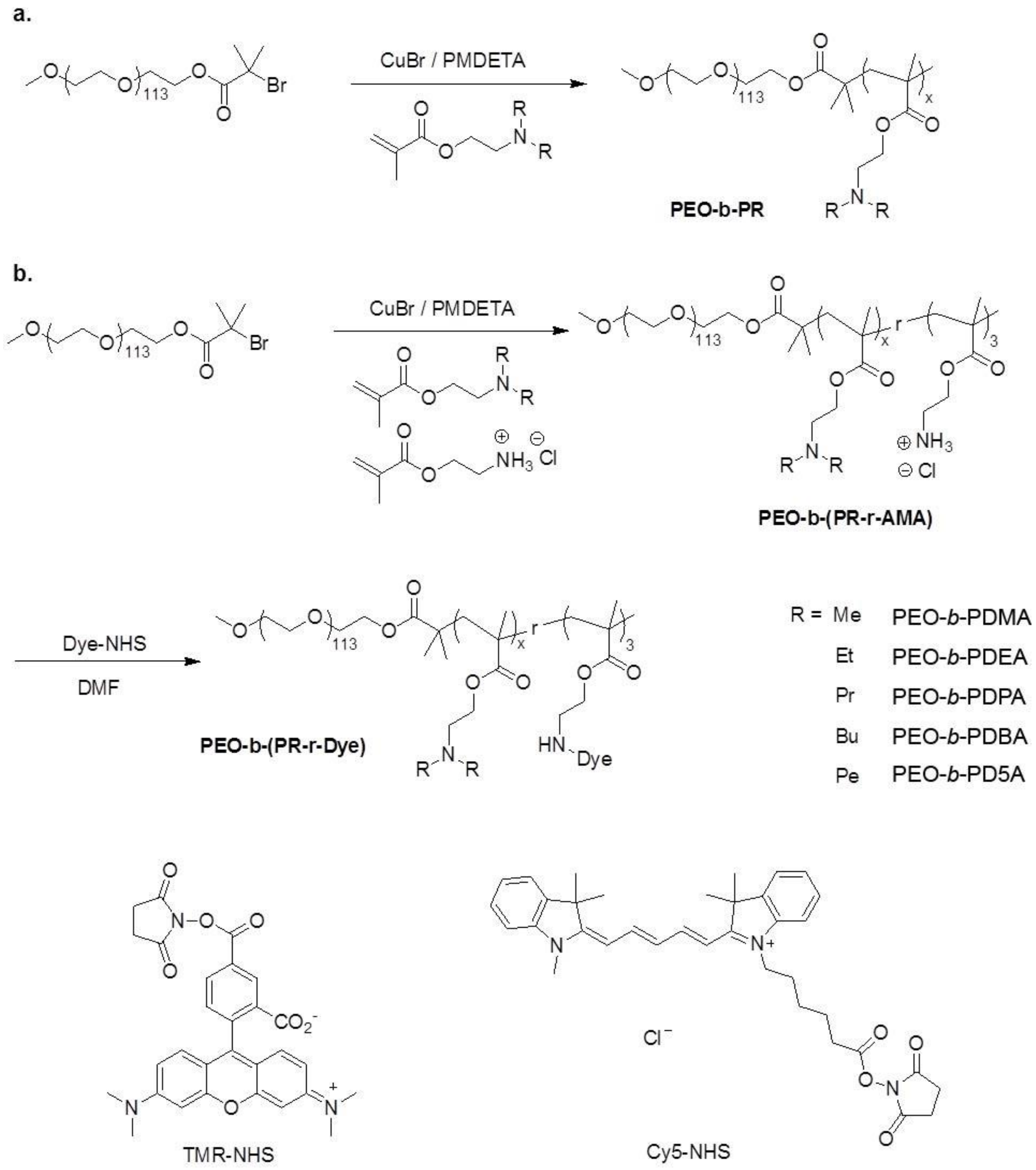

Supplementary Figure 1. Syntheses of (a) PEO-*b*-PR and (b) dye-conjugated PEO-*b*-PR copolymers. Without specific mention, x equals to 80 and the conjugation number of fluorophores per polymer chain was 3. In addition, only fluorescent dye was conjugated to the polymer chains. No quencher molecules were incorporated.

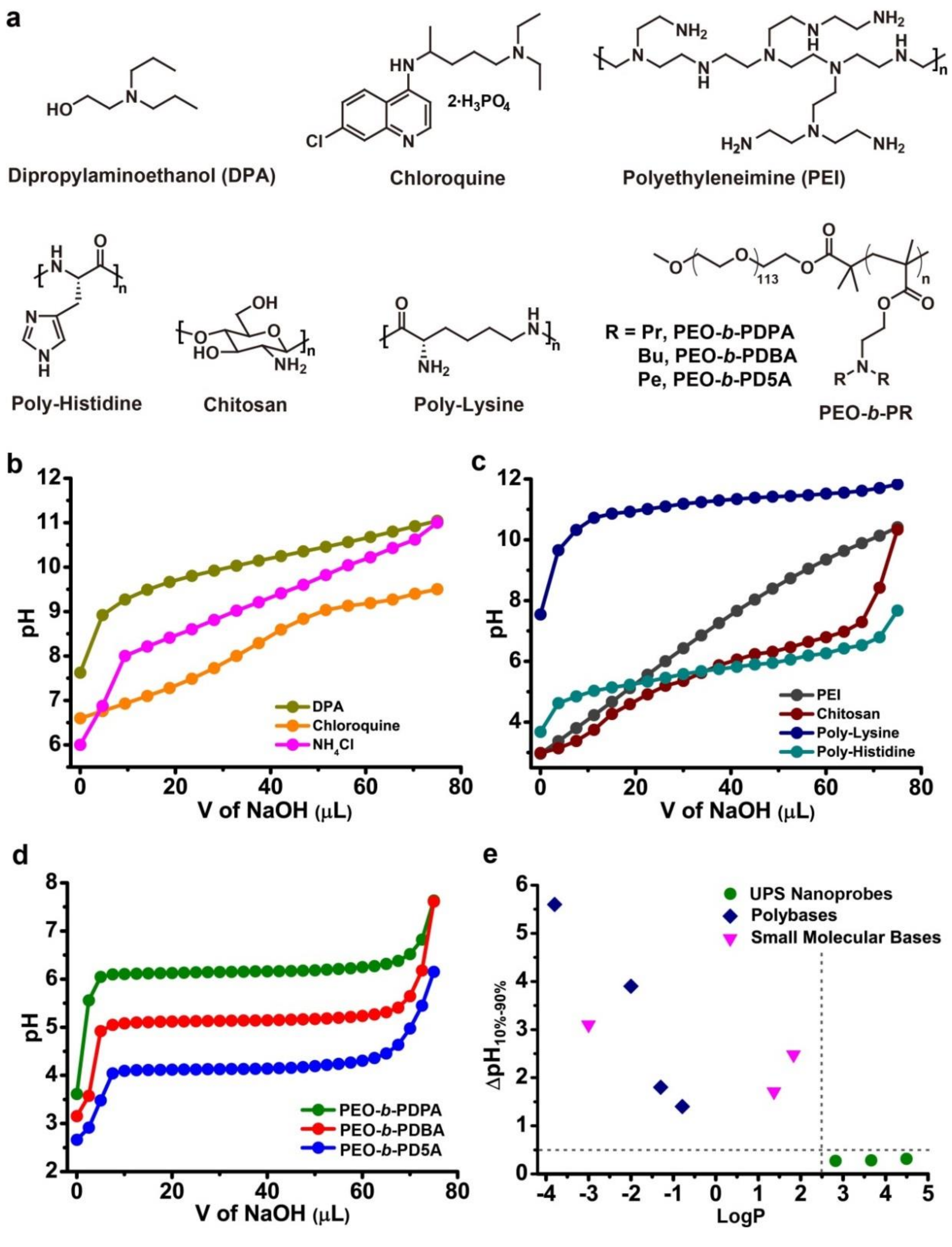

Supplementary Figure 2. a, Structures of applied pH sensors. pH titration curves of small molecular bases (**b**), polymeric bases (**c**) and PEO-*b*-PR block copolymers (**d**). **e**, pH response sharpness as a function of octanol-water partition coefficient (LogP) of small molecular bases (NH_4Cl, Chloroquine and DPA) or repeating unit (neutral form) from commonly used polymeric bases (poly(ethyleneimine), polylysine, chitosan, polyhistidine) and UPS block copolymers (PEO-*b*-PDPA, PEO-*b*-PDBA and PEO-*b*-PD5A).

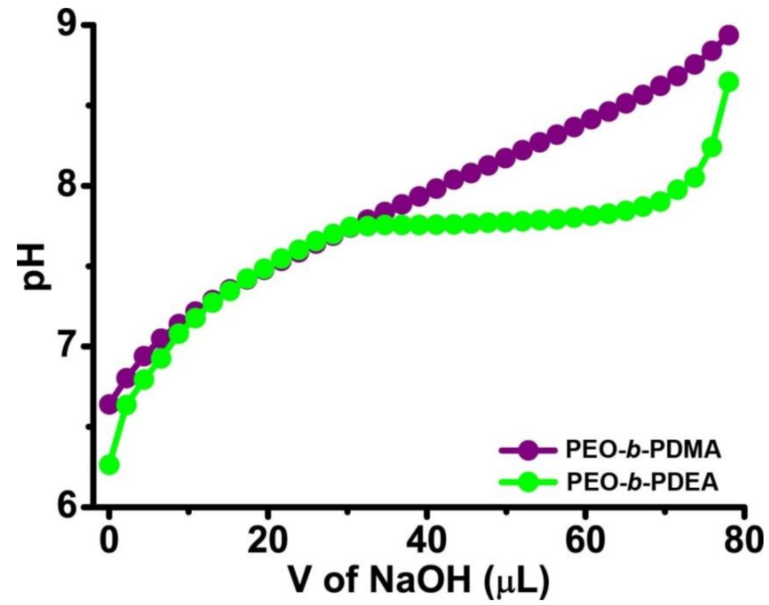

Supplementary Figure 3. pH titration curves of PEO-*b*-PDMA and PEO-*b*-PDEA copolymers. No micelles were formed throughout the titration course of PEO-*b*-PDMA. PEO-*b*-PDEA showed a divergent behavior, where a broad pH response was observed when protonation degree was above 60%, and an ultra-pH responsive behavior below 60%.

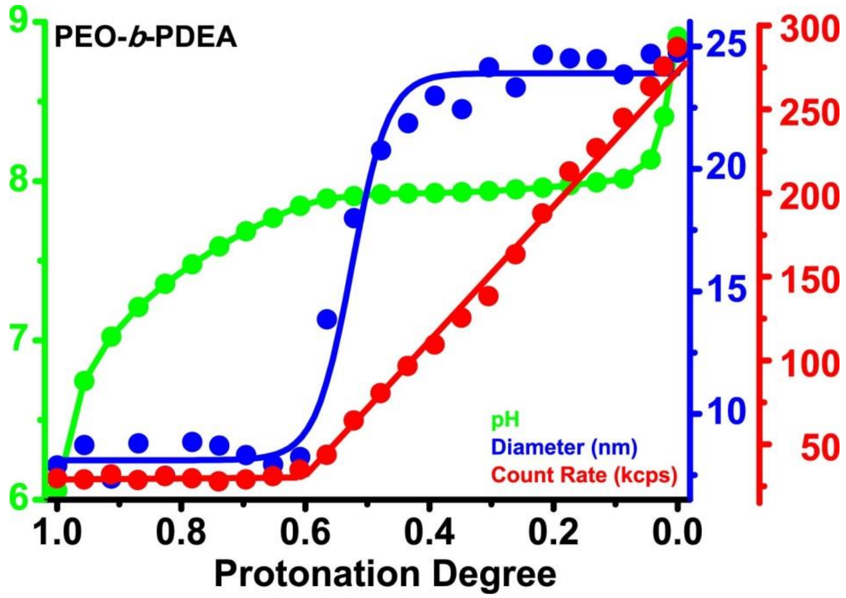

Supplementary Figure 4. Changes in hydrodynamic diameter and light scattering count rates along the pH titration course of PEO-*b*-PDEA copolymer. The sharp pH response did not start until micelle formation, as indicated by the increase of hydrodynamic diameter and count rate.

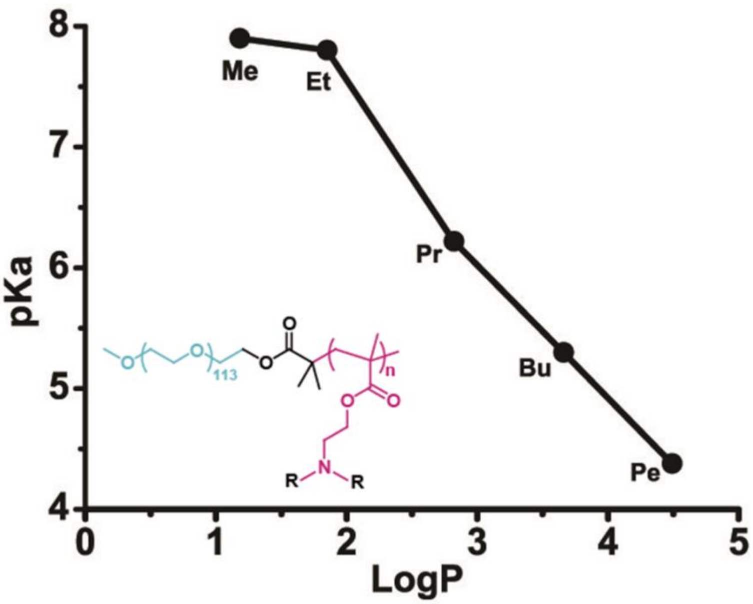

Supplementary Figure 5. pKa values of PEO-*b*-PR copolymers with different terminal groups (R). More hydrophobic side chains lead to lower pKa of the copolymer.

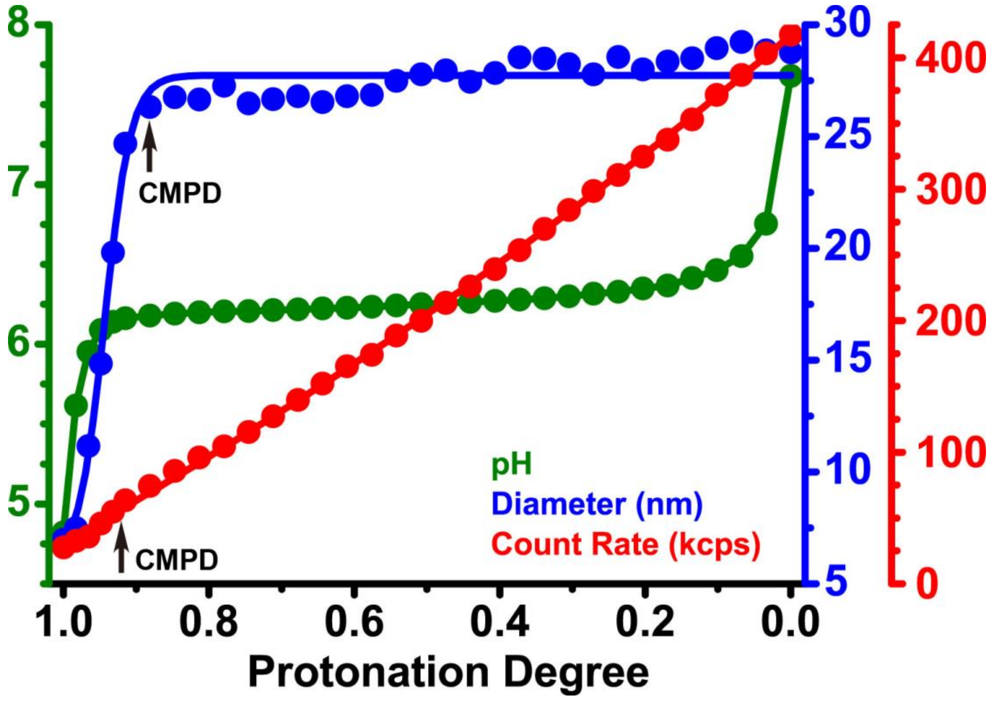

Supplementary Figure 6. Changes in hydrodynamic diameter and light scattering count rates along pH titration course of PEO-*b*-PDPA copolymer.

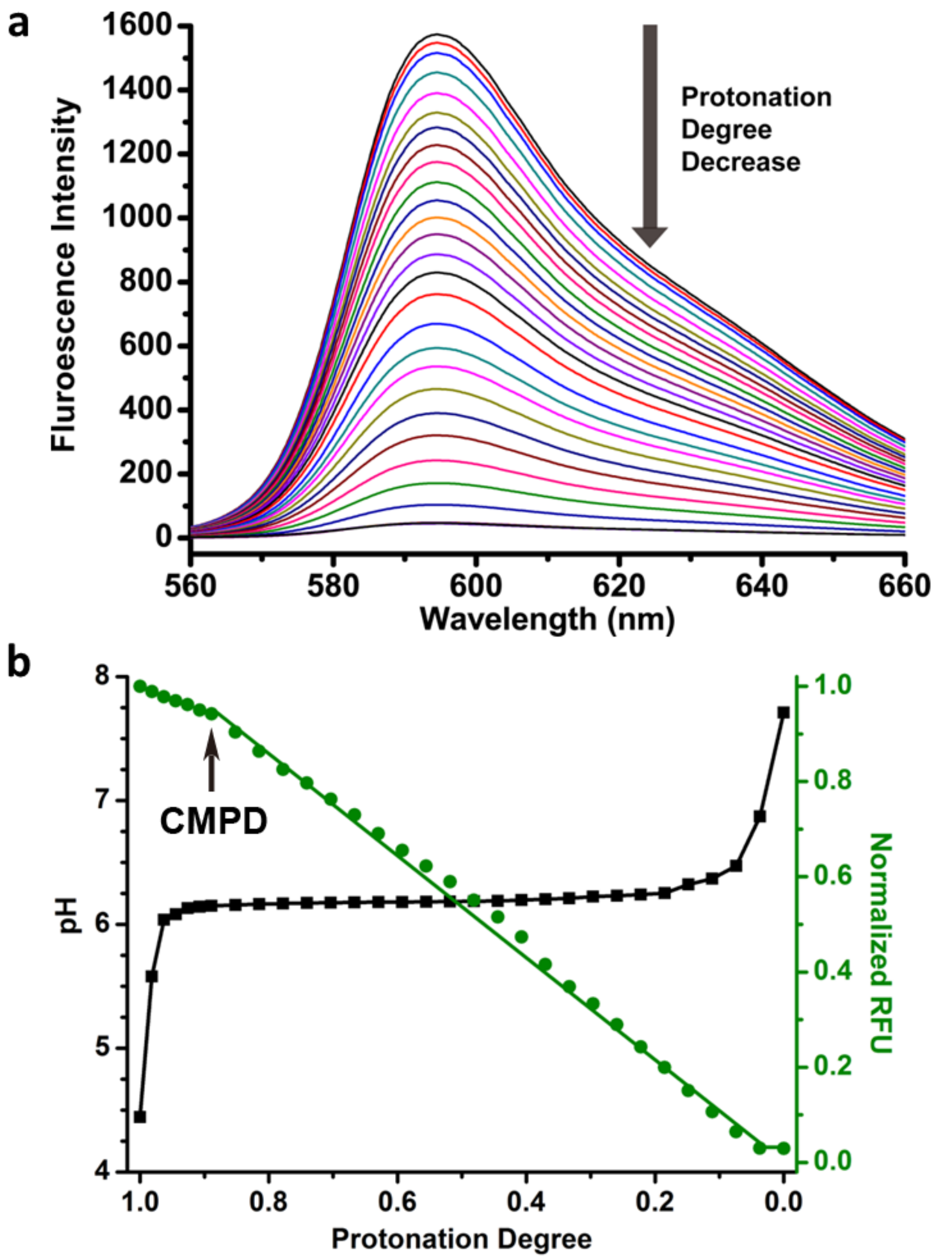

Supplementary Figure 7. a, Measurement of fluorescence intensity of TMR conjugated PEO-*b*-PDPA copolymers along pH titration course. **b**, Plot of pH and fluorescence intensity changes as a function of protonation degree.

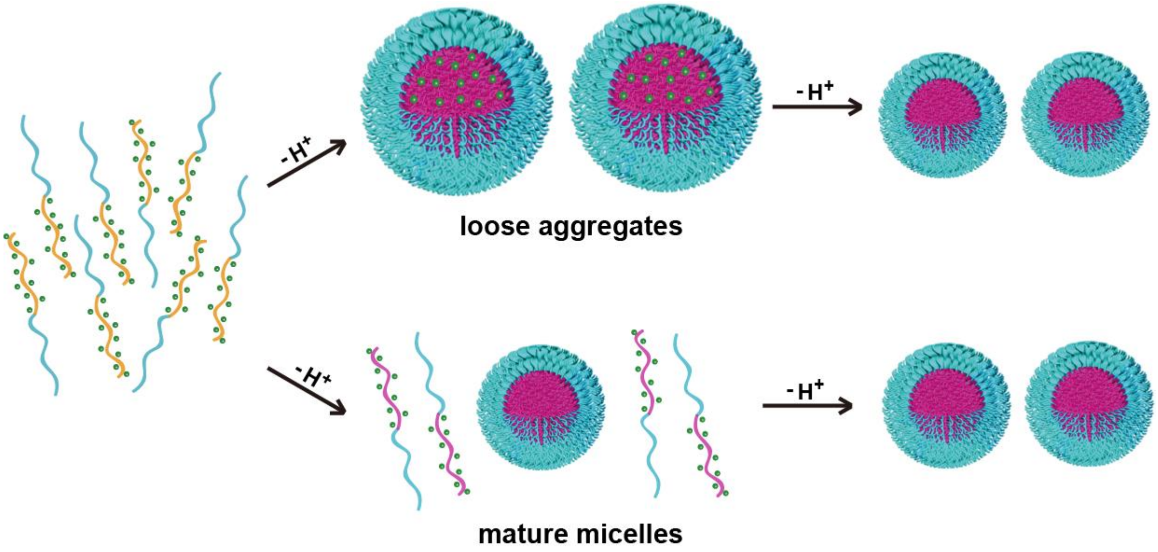

Supplementary Figure 8. Two possible pathways for pH-triggered supramolecular self-assembly. In the graduate model (upper panel), the fully charged unimers are initially deprotonated into positively charged loose aggregates. Upon further neutralization, the loose aggregates are gradually deprotonated, shrinking in size and finally turning into neutral, mature micelles. In the divergent model (lower panel), at any given protonation degree below CMPD, the PEO-*b*-PDPA copolymers exist in either protonated unimers or neutral, mature micelles with different population distributions. It should be noted that the above molecular pathway was experimentally validated by methods (e.g., DLS, ^{1}H NMR) that determine the thermodynamically stable states along the pH titration coordinate. The kinetic pathway of conversion of protonated unimer to neutral, mature micelles may still involve loose aggregates as transient intermediates.

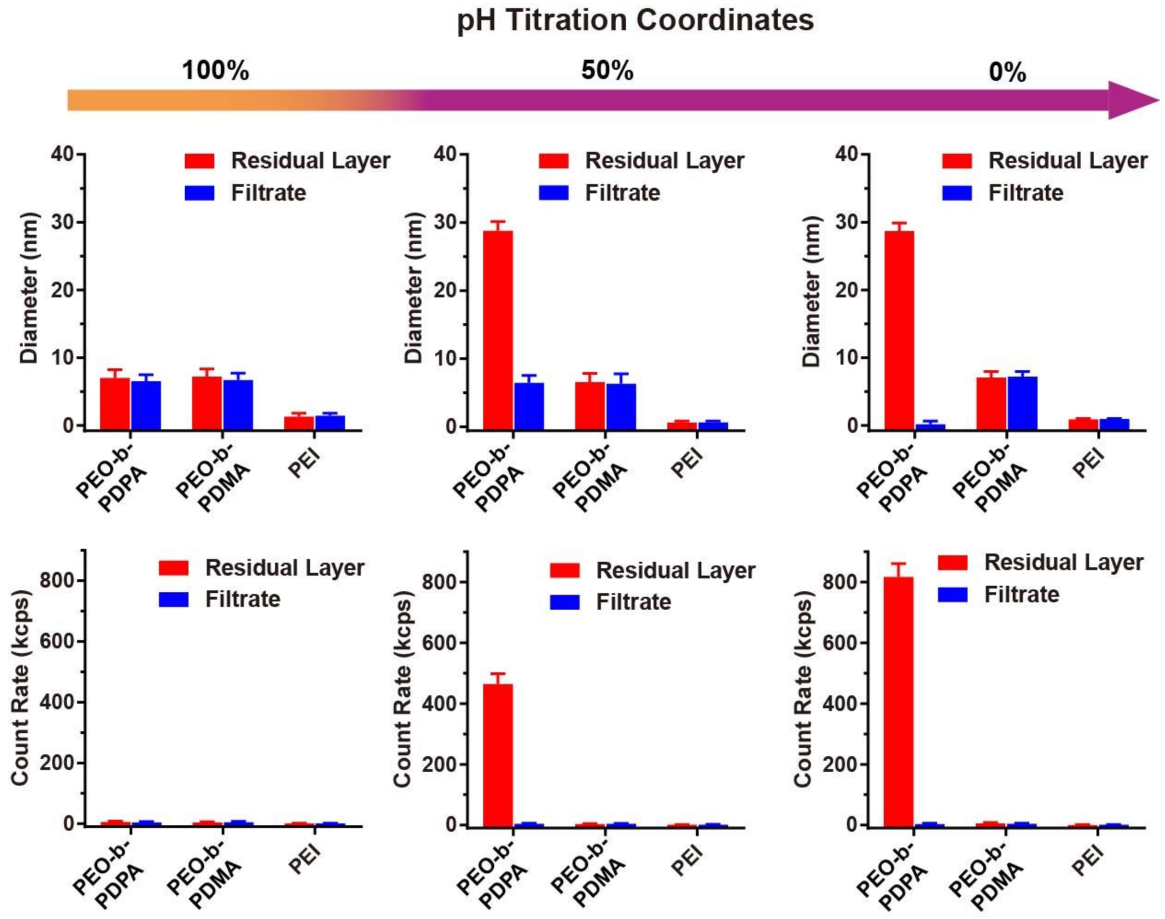

Supplementary Figure 9. Hydrodynamic diameters and light scattering count rates of residual and filtrate layers at three protonation degrees (100, 50 and 0%) of PEO-*b*-PDPA, PEO-*b*-PDMA and PEI.

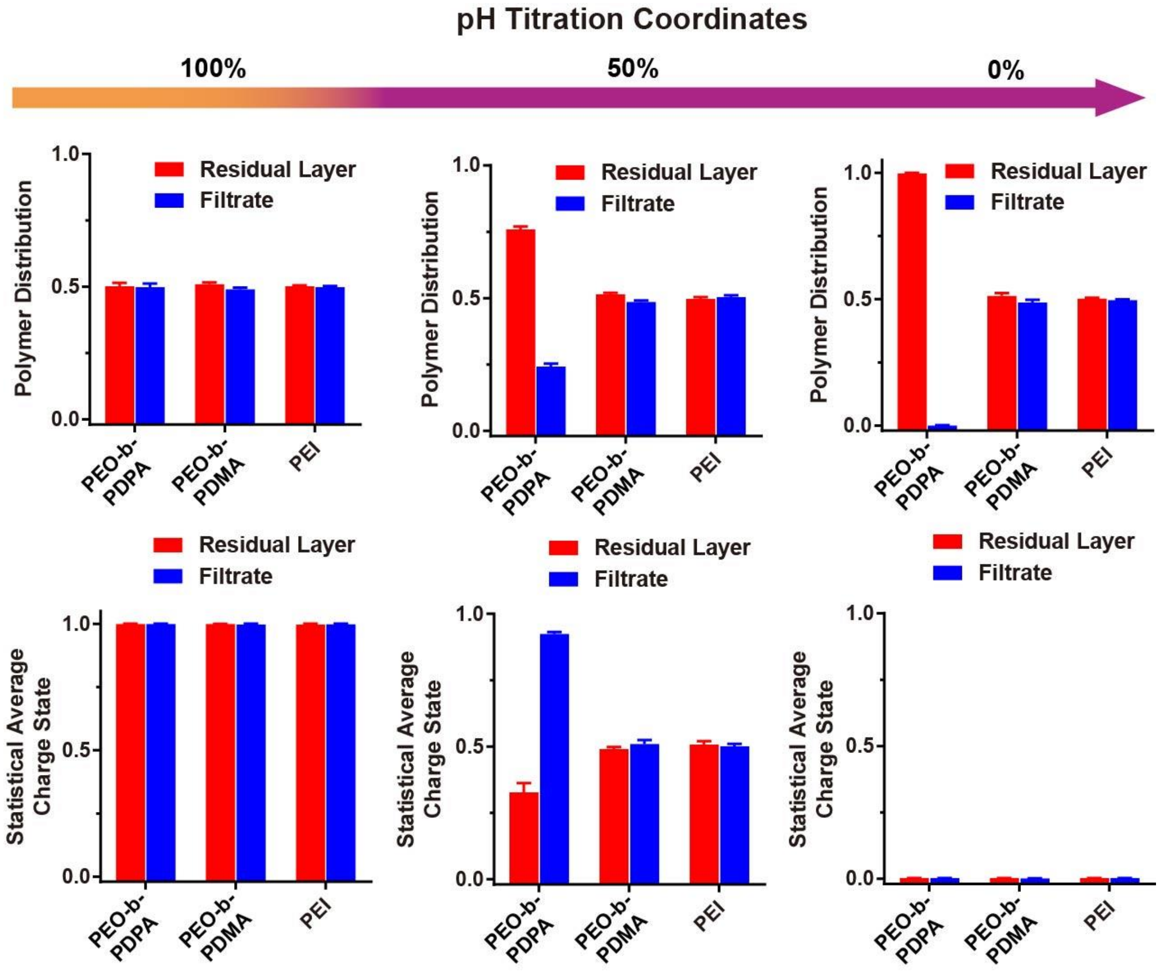

Supplementary Figure 10. Polymer mass and charge state distributions in the residual and filtrate layers at three protonation degrees (100, 50 and 0%) of PEO-*b*-PDPA, PEO-*b*-PDMA and PEI.

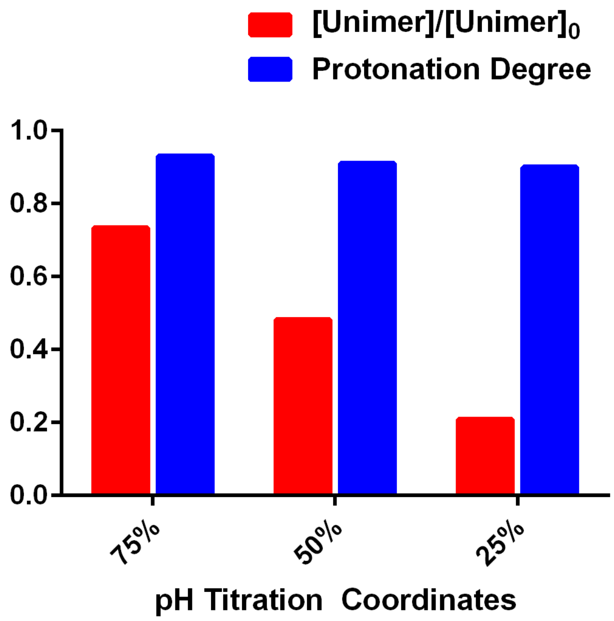

Supplementary Figure 11. Unimer molar fraction ($[Unimer]/[Unimer]_0$) in the filtrate layer and their protonation degrees at different stages of pH titration of PEO-*b*-PDPA.

Dipropylaminoethanol

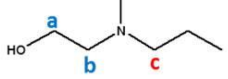

		0%	20%	40%	60%	80%	100%
Chemical Shift	a	3.70	3.74	3.78	3.81	3.85	3.89
	b	2.70	2.83	2.94	3.06	3.19	3.30
	c	2.51	2.64	2.76	2.89	3.03	3.15

PEO-*b*-PDMA

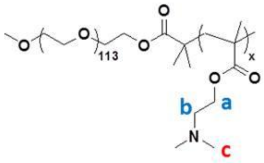

Chemical Shift	a	4.16	4.20	4.24	4.29	4.34	4.40
	b	2.74	2.86	3.01	3.20	3.37	3.58
	c	2.32	2.44	2.57	2.72	2.84	3.01
Integration	a	96.76	101.74	102.94	102.47	101.90	101.03
	b	99.73	103.07	101.03	101.56	103.71	102.09
	c	325.61	325.92	324.15	323.06	324.70	325.48

PEO-*b*-PDPA

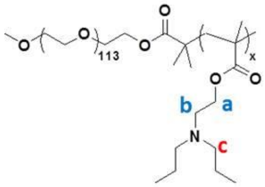

Chemical Shift	a	-	4.420	4.420	4.420	4.423	4.432
	b	-	3.59	3.59	3.59	3.59	3.607
	c	-	3.22	3.22	3.22	3.22	3.24
Integration	a	-	26.21	47.31	63.69	84.70	107.66
	b	-	28.52	46.16	65.67	86.07	108.59
	c	-	55.04	102.16	137.08	181.15	228.15

Supplementary Figure 12. Chemical shifts and peak integrations of different proton signals in DPA, PEO-*b*-PDMA and PEO-*b*-PDPA. All samples were prepared using deuterated D_2O, DCl and NaOD. For PEO-*b*-PDMA and PEO-*b*-PDPA, peak integration was based on proton signals of interests relative to internal reference of PEO segment. The results from proton "c" were included as examples in the main text.

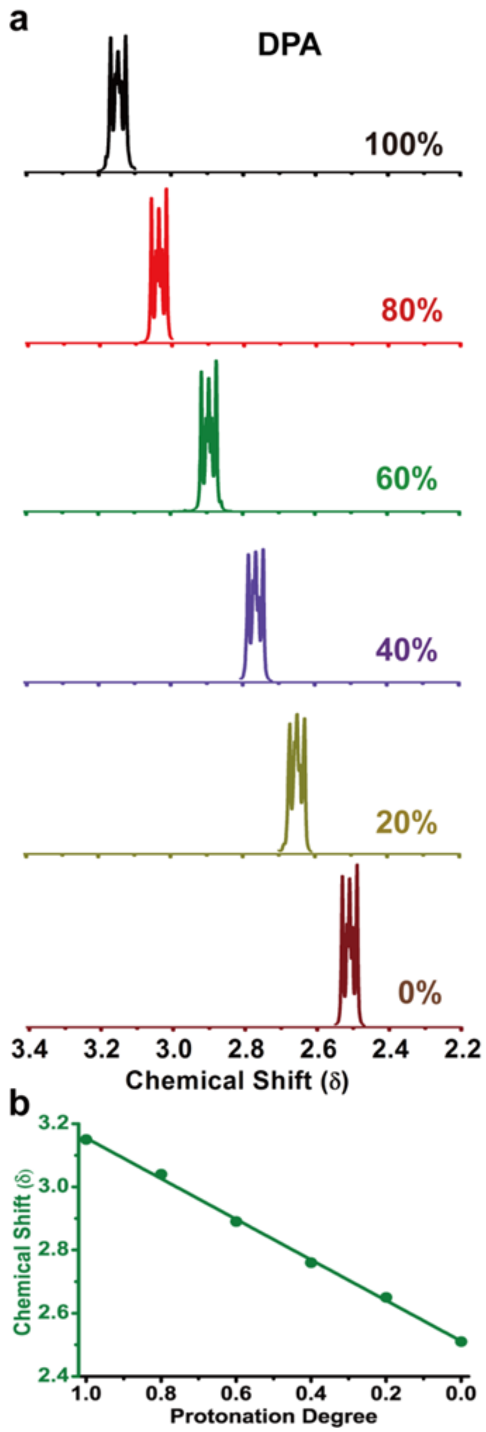

Supplementary Figure 13. Chemical shift of methylene protons (c in Supplementary Fig. 11) of dipropylaminoethanol (DPA) at different protonation degrees.

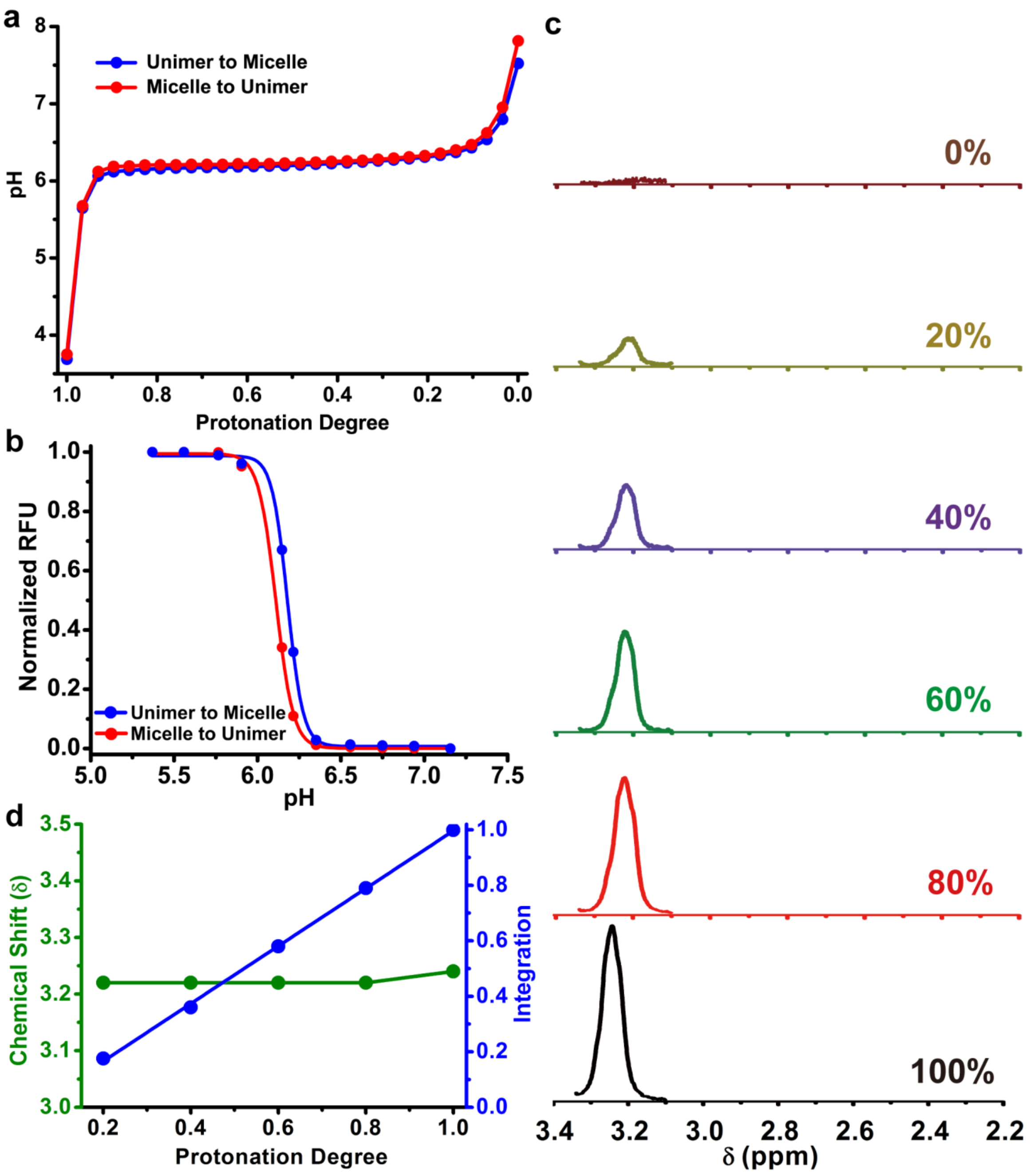

Supplementary Figure 14. Microscopic reversibility of pH titration of PEO-*b*-PDPA copolymers. **a**, pH titration; **b**, fluorescence emission intensity as a function of pH of TMR conjugated PEO-*b*-PDPA block copolymers; **c-d,** ^{1}H NMR spectra of micelle to unimer transitions of PEO-*b*-PDPA copolymers (opposite to the titration direction as shown in Fig. 4b and 4d).

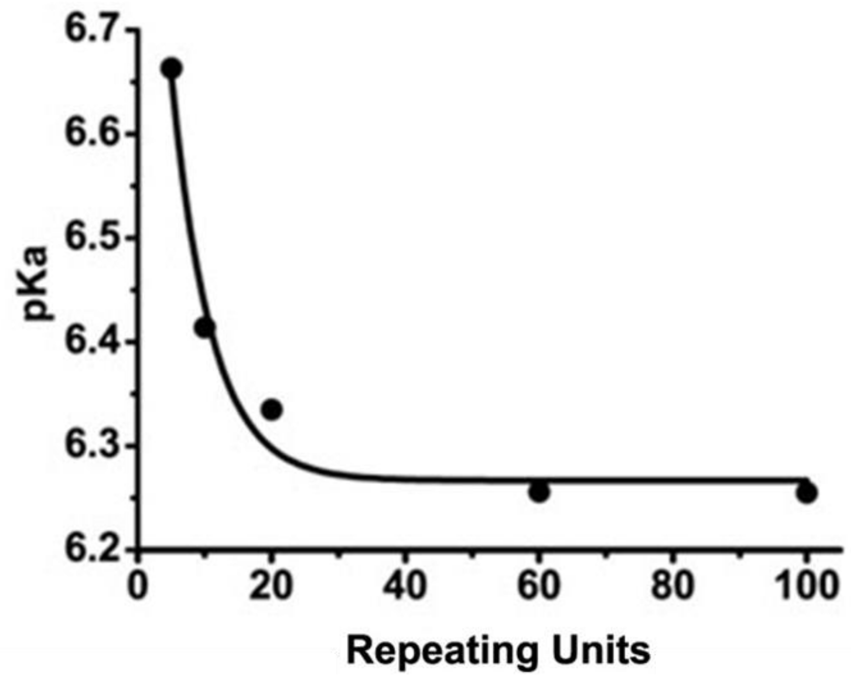

Supplementary Figure 15. pKa of PEO-*b*-PDPA copolymers decreases with increasing polymer chain length. Longer hydrophobic chain length leads to lower pKa.

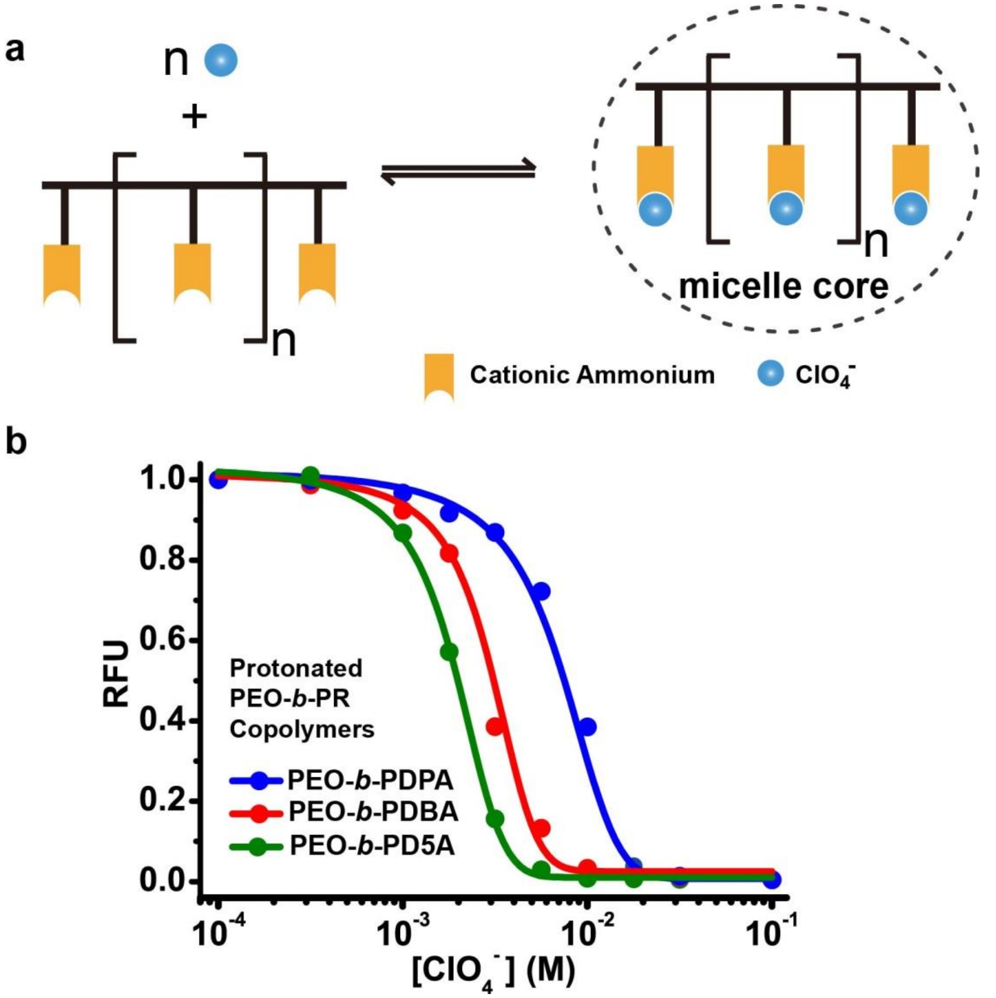

Supplementary Figure 16. a, Schematic illustration of ClO_4^- binding to protonated PEO-*b*-PR block copolymers, which is treated as monovalent ligands binding to multi-site receptors. **b**, Normalized fluorescent intensity of TMR-conjugated cationic PEO-*b*-PR block copolymers as a function of ClO_4^- concentration. Copolymers with more hydrophobic side chains had lower critical concentration necessary for the formation of micelles. In the absence of cooperativity, the transition from bound-free state to fully occupied state will require over 100 fold ligand concentration. The fluorescent on/off transition of dye-conjugated PEO-*b*-PR copolymers requires ~3-fold in ClO_4^- concentrations, indicating positive binding cooperativity.

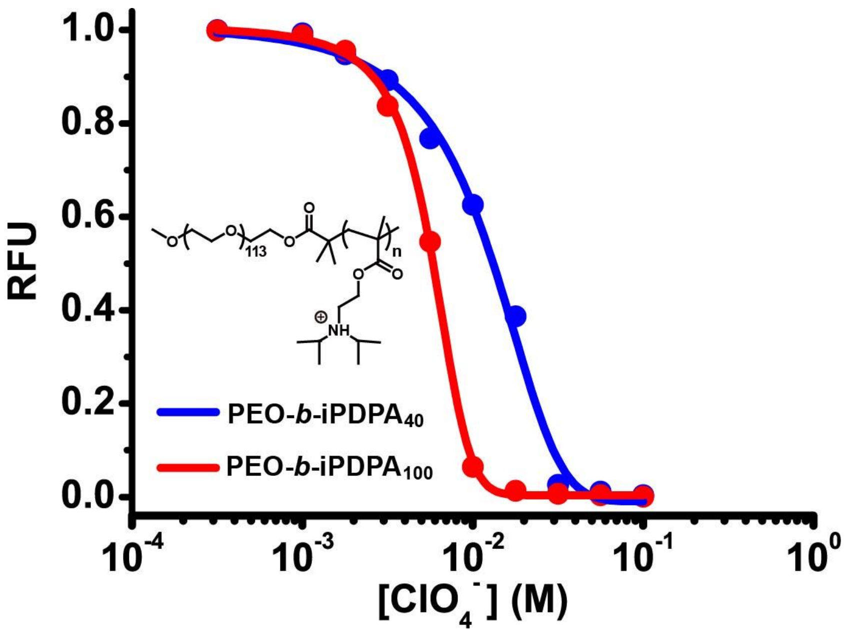

Supplementary Figure 17. Normalized fluorescent intensity of TMR-conjugated cationic PEO-*b*-^{i}PDPA (isopropyl group as terminal alkyl groups) copolymers as a function of ClO_4^- concentration. For positive cooperative binding between monovalent ligands and multi-site receptors, increasing the binding sites per chain will increase binding cooperativity. Sharper on/off transition and increased detection sensitivity as a result of increased hydrophobic chain length confirm the positive cooperativity in the ClO_4^- and cationic PEO-*b*-PR interactions.

Supplementary Table 1. Characterization of PEO-*b*-PR block copolymers.

Copolymer	Yield (%)	$M_{w,GPC}$ ($\times 10^{-4}$ D)[a]	$M_{n,GPC}$ ($\times 10^{-4}$ D)[a]	PDI[a]	Repeating units In the PR block	$M_{n,1H\ NMR}$ (x 10^{-4} D)[b]
PEO-*b*-PDMA	89	2.27	1.85	1.23	90	1.92
PEO-*b*-PDEA	88	2.32	1.93	1.20	88	2.13
PEO-*b*-PDPA	87	2.48	2.05	1.21	80	2.20
PEO-*b*-PDBA	77	2.79	2.35	1.19	73	2.26
PEO-*b*-PD5A	71	3.12	2.61	1.20	83	2.73

[a] Number-average (Mn), weight-average molecular weight (Mw) and polydispersity index (PDI) (PDI=Mw/Mn) were determined by GPC using THF as the eluent. [b] Determined by ^{1}H-NMR.

Supplementary Table 2. Characterization of PEO-*b*-nPDPA block copolymers with different hydrophobic chain length

Copolymer	$M_{w,GPC}$ ($\times 10^{-4}$ D)[a]	$M_{n,GPC}$ ($\times 10^{-4}$ D)[a]	PDI[a]	Repeating units In the PR block	$M_{n,1H\ NMR}$ (x 10^{-4} D)[b]
PEO-*b*-$nPDPA_5$	0.87	0.66	1.32	6	0.63
PEO-*b*-$nPDPA_{10}$	1.07	0.83	1.29	10	0.72
PEO-*b*-$nPDPA_{20}$	1.32	1.09	1.22	22	0.98
PEO-*b*-$nPDBA_{60}$	2.29	2.01	1.14	64	1.86
PEO-*b*-$nPDPA_{100}$	3.33	2.71	1.23	98	2.59

[a] Number-average (Mn), weight-average molecular weight (Mw) and polydispersity index (PDI) (PDI=Mw/Mn) were determined by GPC using THF as the eluent. [b] Determined by ^{1}H-NMR.

Supplementary Discussion

Development of the cooperative deprotonation model

We adopted an allosteric model to describe the cooperative deprotonation process (Fig. 5a). The sequential neutralization of fully protonated polymers can be characterized by a series of microscopic *Ki*, which corresponds to the *i*th dissociation constant of the polyatomic acids.

$$H_{n-i+1}A \underset{}{\overset{K_i}{\rightleftharpoons}} H_{n-i}A + H^+ \qquad (1)$$

Cooperative parameters

In the simplest case of dibasic acid (n=2), the neutralization of protons can be characterized by two microscopic dissociation constant K_1 and K_2 as defined by Supplementary Equation 2 and 3, where 2 and ½ are statistical factors[1].

$$2K_1 = \frac{[HA^-][H^+]}{[H_2A]} \qquad (2)$$

$$1/2K_2 = \frac{[A^{2-}][H^+]}{[HA^-]} \qquad (3)$$

At molecular level, the cooperativity of dibasic acid dissociation process can be described by the interaction parameter α, which is defined as[2]:

$$\alpha = K_2 / K_1 \qquad (4)$$

For the dissociation of polyatomic acid as in our system, a series of cooperative parameters α are defined by Equation 1 as shown in the main text. The apparent dissociation constant K is defined as geometric mean of individual dissociation constants[3] (Equation 2 or Supplementary Equation 5).

$$K = \sqrt[n]{K_1 K_2 \ldots\ldots K_n} \qquad (5)$$

Assuming identical cooperative parameter α for each dissociation step (Supplementary Equation 6), Supplementary Equation 5 can be simplified as Equation 3 or Supplementary Equation 7. This equation allows the theoretical-experimental correlation between the microscopic cooperative parameter α and macroscopically measurable pKa (logarithmic *K*). For polymeric polyatomic acid, the pKa can be defined as the pH where 50% of the ionizable amines are protonated[4-6]. The theoretical modeling suggests that cooperative dissociation of fully protonated PEO-*b*-PR with more ammonium groups per polymer chain will have higher *K* (or *K*a) and lower pKa.

$$\alpha = \alpha_1 = \alpha_2 = \bullet\bullet\bullet\bullet\bullet\bullet = \alpha_{n-1} \qquad (6)$$

$$K = \sqrt{\alpha^{n-1}}\, K_1 \qquad (7)$$

Hill Coefficient. Practically, Hill plot is used to evaluate the allosteric cooperativity. First, the sequential neutralization of fully protonated UPS nanoprobes are described in dissociation isotherms. Here we use (pH - pKa) as the x-axis and protonation degree (θ_A) as the y-axis to plot the dissociation isotherms. In this case, the point at which 50% of tertiary amines are protonated occurs at 0 on the x-axis[7]. Increase of pH leads to dissociation of protons, corresponding to the decreased θ_A as shown in Figure 5b, d.
For classic allosteric binding of monovalent ligand B to multi-site receptors A….A , the dependence of the binding site occupancy θ_A can be generalized as[7]:

$$\theta_A = \frac{(K[B])^n}{1 + (K[B])^n} \qquad (8)$$

Hill plot is based on a rearranged version of this equation to characterize the strength of cooperative binding interactions by ploting log(θ_A /(1- θ_A)) versus logarithmic ligand concentration Log[B][7]:

$$\mathrm{Log}\left(\frac{\theta_A}{1 - \theta_A}\right) \approx n\,\mathrm{Log}(K'[B]) \qquad (9)$$

The deprotonation is opposite process of protons binding to tertiary amines, which means the K=1/K′ . So the cooperativity in allosteric deprotonation of fully charged PEO-*b*-PR copolymers can be quantified by plotting log(θ_A /(1- θ_A)) versus (pKa-pH) in a Hill plot:

$$\mathrm{Log}\left(\frac{\theta_A}{1 - \theta_A}\right) \approx n(\mathrm{pKa} - \mathrm{pH}) \qquad (10)$$

The Hill coefficient n_H, corresponding to the slope of this plot measured at 50% saturation, is a useful parameter to quantify the cooperativity strength. The allosteric systems with positive cooperativity have the number of binding sites n as the limit of n_H. In the absence of cooperativity, the n_H is equal to 1. Positive cooperaitvity gives a slope larger than 1 (n_H >1), which corresponds to narrower ligand concentration range nacessary for receptors to go from bound free state to fully occupied state. The Hill plot modeling suggests that polyatomic acids with more ammonium groups per polymer chain will have higher n_H and sharper pH transition.

Supplementary Methods

Materials

N-Hydroxysuccinimidal ester of tetramethyl rhodamine (NHS-TMR) was purchased from the Invitrogen Company. Cy5-NHS ester was purchased from the Lumiprobe Corporation. PEO macroinitiator, MeO-PEO_{114}-Br, was prepared from 2-bromo-2-methyl propanoyl bromide and MeO-PEO_{114}-OH according to the procedure in literature. Monomers such as 2-(dimethylamino)ethyl methacrylate (DMA-MA), 2-(diethylamino)ethyl methacrylate (DEA-MA) and 2-aminoethyl methacrylate (AMA) were purchased from Polyscience Company. The 2-(dibutylamino) ethyl methacrylate (DBA-MA) monomer was synthesized following a previous publication[8]. AMA monomer was recrystallized twice with isopropanol and ethyl acetate (3:7) before use. Bromopropane, bromopentane, ethanolamine, methacryloyl chloride and sodium salts were purchased from Sigma-Aldrich. Other solvents and reagents were used as received from Sigma-Aldrich or Fisher Scientific Inc.

Syntheses of methacrylate monomers

Methacrylate monomers were synthesized following a published method. [2] Synthesis of 2-(dipropylamino) ethyl methacrylate (DPA-MA) is described here as an example. First, ethanolamine (12.2g, 0.2 mol) and bromopropane (49.2 g, 0.4 mol) were dissolved in 400 mL acetonitrile, and Na_2CO_3 (53.0 g, 0.5 mol) was added to the solution. After overnight reaction, the solution was filtered to remove the precipitated NaBr salt and extra Na_2CO_3. CH_3CN solvent was removed by rotovap. The resulting residue was distilled *in vacuo* (40~45 ℃ at 0.05 mm Hg) as a colorless liquid to obtain 2-(dipropylamino) ethanol. Then 2-(dipropylamino) ethanol (21.3g, 0.1 mol), triethylamine (10.1 g, 0.1 mol), and inhibitor hydroquinone (0.11g, 0.001mol) were dissolved in 100 mL CH_2Cl_2 and methacryloyl chloride (10.4g, 0.1 mol) was added dropwise into a three-neck flask. The solution was refluxed overnight. After reaction, the solution was filtered to remove the precipitated triethylamine-HCl salts, and CH_2Cl_2 solvent was removed by rotovap. The resulting residue was distilled *in vacuo* (47-53 ℃ at 0.05 mm Hg) as a colorless liquid. After synthesis, the monomer was characterized by ^{1}H-NMR. All the NMR spectra were obtained in $CDCl_3$ using tetramethylsilane (TMS) as the internal reference on a Varian 500MHz spectrometer. The characterization of the DPA methacrylate monomers is as follows:

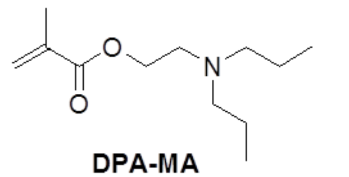

DPA-MA

2-(Dipropylamino) ethyl methacrylate (DPA-MA)

^{1}H NMR (TMS, $CDCl_3$, ppm): 6.10 (br, 1H, C*H*H=C(CH_3)-), 5.54 (br, 1H, CH*H*=C(CH_3)-), 4.07 (t, 2H, -OC$H_2$$CH_2$N-), 3.01 (t, 2H, -O$CH_2CH_2$N-), 2.68 (t, 4H, -N(C$H_2$$CH_2$$CH_3$)$_2$, 1.94 (s, 3H, CH_2=C(CH_3)-), 1.44 (m, 4H, -N(CH_2C$H_2$$CH_3$)$_2$), 1.01(t, 6H, -N($CH_2$$CH_2CH_3$)$_2$)

Syntheses of dye conjugated PEO-*b*-PR block copolymers

AMA monomer was incorporated in the copolymers for the conjugation of dyes (Scheme S1b) following procedures reported previously[3]. Synthesis of PEO-*b*-(PR-r-AMA) copolymers followed the procedure described above. Three primary amino groups were introduced into each polymer chain by controlling the feeding ratio of AMA monomer to the initiator (ratio = 3). In a representative procedure, PEO-*b*-(PR-r-AMA) (50mg) was dissolved in 2 mL DMF. Then the NHS-ester (3.0 equivalence for TMR-NHS, RhoG or Cy5-NHS) was added. After overnight reaction, the copolymers were purified by preparative gel permeation chromatography (PLgel Prep 10 m 10E3 Å 300×250 columns by Varian, THF as eluent at 5 mL/min) to remove the free dye molecules. The produced PEO-*b*-(PR-*r*-Dye) copolymers were lyophilized and kept at -20 ℃ during storage.

TEM and DLS characterization

Samples for TEM and DLS analyses were prepared *in situ* by pH titration. The morphology and size of nanoparticles were characterized by transmission electron microscopy (TEM, FEI Tecnai G2 Spirit Biotwin model). Hydrodynamic diameter (D_h) and scattering count rates were determined by dynamic light scattering (DLS, Malvern Nano-ZS Model, He-Ne Laser, λ=633 nm).

Fluorescence characterization

The fluorescence emission spectra were obtained on a Hitachi fluorometer (F-7500 model, Tokyo, Japan). The fluorescent images of PEO-*b*-PDBA-RhoG and Lysosensor Green solutions at different pH values (200 μg/mL for each sample) were obtained using the Maestro imaging system (CRI, Inc., Woburn, MA) with a proper band pass excitation filter and long-pass emission filter according to the instrument manual. All measurements were conducted at room temperature.

Perchlorate Anion Sensing

TMR-conjugated PEO-*b*-PR copolymer stock solutions were prepared following a solvent evaporation method as previously published[8]. In the example of PEO-*b*-PDPA micelle solution, 40 mg of the dye-conjugated copolymer was first dissolved in 2 mL THF and then added into 8 mL distilled water dropwise under sonication. The THF was removed through ultrafiltration with (100 kD) membrane for several times. Then the distilled water was added to adjust the polymer concentration to 5.0 mg/mL as a stock solution.

TMR conjugated PEO-*b*-PDPA stock solution (2 mL) was first diluted to 2.0 mg/ml by addition of DI water. 1.0 M HCl was added to adjust the pH to 4.0 where the micelles dissociated into fully protonated unimers. ClO_4^- titration was carried out by adding small volumes of 1.0 M or 10.0 M $NaClO_4$ solution under stirring. The fluorescence of dye-conjugated polymer solution was measured at different ClO_4^- concentration. Titration of

other protonated PEO-*b*-PR copolymers followed similar procedures. The Relative Fluorescence Unit (RFU) was normalized as F/F_{max} where F was the fluorescence intensity of dye-conjugated polymer solution at difference ClO_4^- concentration and F_{max} was the maximum fluorescence intensity without ClO_4^-.

Supplementary References

1 Ercolani, G., Piguet, C., Borkovec, M. & Hamacek, J. Symmetry numbers and statistical factors in self-assembly and multivalency. *J. Phys. Chem. B,* **111**, 12195-12203 (2007).

2 Connors, K. A., Paulson, A. & Toledo-Velasquez, D. Complexing of. alpha.-cyclodextrin with sym-4, 4'-disubstituted biphenyls. J. Org. Chem., **53**, 2023-2026 (1988).

3 Chang, R. *Physical chemistry for the chemical and biological sciences*. (University Science Books, 2000).

4 Kokufuta, E., Terada, T., Tamura, M., Suzuki, S. & Harada, K. Potentiometric titration behavior of polylysine and copolymer of lysine with alanine prepared by thermal polycondensation. *Arch. Biochem. Biophys.* **196**, 23-32 (1979).

5 Borkovec, M., Koper, G. J. & Piguet, C. Ion binding to polyelectrolytes. *Curr. Opin. Colloid Interface Sci.* **11**, 280-289 (2006).

6 Koper, G. J. & Borkovec, M. Proton binding by linear, branched, and hyperbranched polyelectrolytes. *Polymer* **51**, 5649-5662 (2010).

7 Hunter, C. A. & Anderson, H. L. What is cooperativity? *Angew. Chem. Int. Ed.,* **48**, 7488-7499 (2009).

8 Zhou, K. *et al.* Tunable, Ultrasensitive pH - Responsive Nanoparticles Targeting Specific Endocytic Organelles in Living Cells. *Angew. Chem.Int.Ed.,* **50**, 6109-6114 (2011).
